# Forecasting auditor’s going concern opinion using with hybrid robust machine learning model

**DOI:** 10.1371/journal.pone.0345071

**Published:** 2026-03-20

**Authors:** Uğur Ejder, Alpaslan Yaşar

**Affiliations:** 1 Adana Alparslan Türkeş Science and Technology University, Department of Information Technology, Adana, Turkey; 2 Adana Alparslan Türkeş Science and Technology University, Department of Business, Adana, Turkey; Khalifa University, UNITED ARAB EMIRATES

## Abstract

The importance of forecasting company bankruptcies makes the auditor’s reporting of the going concern opinion (GCO) a focal point for interested parties. Therefore, researchers have recently turned to predicting GCO using various machine learning (ML) methods. The aim of this research is to propose a novel hybrid model that integrates ML models to enhance the prediction accuracy of the system. We use a combination of traditional (classical) and hybrid ML approaches to identify the superior model among 30 models based on empirical data of Turkish companies listed on Borsa Istanbul (BIST) for the period 2017–2021. Given that the distribution of classes in the analysed dataset is balanced, it can be confidently stated that the research is reliable. The ML models are selected in accordance with the non-linear system since the equation system under consideration is the non-linear system. To minimise deviations and errors caused by distribution and fragmentation, the k-fold method is used to separate the training and test data sets. The experimental results show that the Random Forest based AdaBoost hybrid model outperforms traditional and other hybrid ML models in terms of accuracy by 89%.

## 1. Introduction

Predicting bankruptcy is a major problem in accounting and financial decision-making [1–3]. The importance of predicting company bankruptcies draws the attention of information users to the auditor’s going concern opinion (GCO), which addresses whether there is a material uncertainty about an entity’s ability to continue as a going concern. The auditor’s opinion on going concern relates to whether the entity can continue as a going concern for the foreseeable future, generally for 12 months from the end of the period. This opinion, included in the auditor’s report, which is an important part of the company’s financial report, provides valuable information to stakeholders such as investors, creditors, and regulators about the financial health and sustainability of the company.

International Standards on Auditing (ISA) No. 570 (Going Concern) requires the auditor to express an unqualified, qualified, or adverse opinion on going concern when it is not appropriate to use the going concern basis of accounting (ISA 570, Paragraphs 21–23). However, the recent audit reporting failures that resulted in the bankruptcy of the client entity without a GCO have increased public interest in the GCO [4–6]. Considering the potential adverse effects of audit reporting failures, anticipating the uncertainties related to going concern may enable information users to make the right decisions. Therefore, researchers have developed financial models to estimate GCO, but there is mixed evidence on the effectiveness of these models [7,8]. Therefore, it is important to estimate the auditor’s GCO.

Early studies in the literature on GCO prediction mostly used regression-based techniques and have found a variety of both financial and non-financial determinants [9]. In recent studies, machine learning (ML) methods (artificial neural network-ANN, support vector machine-SVM, random forest-RF, decision trees) are used due to the restrictive assumptions of traditional statistical methods (logit, discriminant, probit). Existing studies using ML methods are generally conducted in US (e.g., [4,9,10] and Taiwan (e.g., [2,6,11–13]) samples and using ANN, SVM and CART methods.

This study aims to build a robust model of the auditor’s GCO decision framework based on optimal hybrid ML modelling. Due to the non-linear nature of the system of equations to be solved, a hybrid system with regression models was not used to create the auditor opinion decision support model. In the literature, it is not clearly stated whether the majority of the systems of equations that are solved are linear or non-linear. The linear or non-linear equation system of GCO has not been fully investigated, although it is a widely used ML technique. Another important point is to deal with class imbalance. This is because it can make the auditor think that the majority class is more important than other classes. This can make the auditor say that the company will continue to do well, even when it is not [14]. In this respect, our study fills an important gap in the literature.

In this study, we use data on liquidity, solvency, turnover, and profitability ratios, as well as firm size and audit firm type, which are commonly used in previous literature to determine auditor’s GCO. There are various approaches that can be selected in order to carry out the evaluation of accuracy. Simply splitting the target dataset into a training and a test subset is one of the oldest – and still very useful – methods [15]. Typically, 70 per cent of the data is used to train the model and the rest is used to test the result. This method yields valuable information about accuracy. However, the use of random selection to divide the reference data into training and test groups has some shortcomings. As a result, a different random division may produce a different trained model and a different resulting map. When classification is performed on a given dataset, the result will be highly influenced by the reliability of the training dataset. Let’s assume that there are inconsistencies in the selected training set and that the result generated won’t be very reliable. The k-fold technique has been used to overcome the problem of inconsistent data sets in this study.

This study aims not only to apply existing machine learning algorithms to a specific dataset, but also to design a comparative and interpretable framework that identifies how algorithmic diversity, community composition, and feature interaction patterns shape ongoing business forecasts. By analyzing performance consistency and feature importance across model families, the study provides methodological insight into why certain architectures outperform others in financial auditing contexts. This framework can be generalized to similar decision support systems where interpretability and stability are critical.

The study aims to contribute to research in several ways. The main contributions of our study include:

- It has been noted in the literature that hybrid models tend to perform better in predicting the future. In our extensive research, we find that while the hybrid models are more successful in making predictions than the traditional methods, they are also been less successful. But predictive success increases even more when the right approach is taken.
- In general, financial forecasting studies have favoured either regression models or hybrid models based on regression models. However, these studies do not mention whether the system of equations to be solved is linear or non-linear [14]. The system solved in the study was mentioned and it was explained why such hybrid models had been created. This confirmed the reliability of the study.
- In this study, a comprehensive comparative environment has been created and this study aims to explain in which situation a better-predicted score is achieved. In the literature, many studies do not provide sufficient information about the balance states of the datasets.

The remainder of this paper is organized as follows: Section 2 provides a review of studies that use ML methods to estimate the auditor’s GCO. In Section 3, we explain the dataset used in this study and the design of the hybrid and traditional models. Details of the experimental results are described in section 4. Section 5 presents conclusions.

## 2. Literature review

The importance of accurately assessing whether there is going concern uncertainty has increased researchers’ interest in estimating GCO. Studies have used traditional statistical and/or ML methods to predict GCO [16,10].

Early studies to predict GCO included logistic regression analysis [10, 16–22], multiple discriminant analysis [7,18,23–25] and probit analysis [25–27]. However, traditional statistical methods have the disadvantage of some restrictive assumptions (linearity, normality, independence among input variables), which may lead to misjudgments and higher error rates in the going concern estimation [2,6,11,12]. Therefore, in recent years, GCOs have been estimated using ML methods with higher prediction accuracy and lower error rates, which are at least considered complementary to traditional statistical methods [2,11,14]. In these studies, ML methods such as artifical neural network (ANN) [4,6,9,11,12,16], support vector machine (SVM) [5,6,11], decision trees [4,11–13,28] random forest [2,10] have started to be adopted.

The existing studies, as shown in Table 1, are generally conducted on US and Taiwanese companies and use ANN, SVM, and CART methods. Our study extends the existing literature by using hybrid ML techniques (RF, XGB, GBM, MPL, KNN, SVM) to predict auditor GCO in Turkey, a developing European country.

**Table 1:** Literature table of previous studies using machine learning methods on GCO prediction.

No	Author	Dataset	Method
1	[29]	40 GCO and 40 non-GCO, 1982-1987, United States	Artificial Neural Networks (ANN), Logistic Regression (LR)
2	[16]	45 GCO and 45 non-GCO, 1990-1991, United States	ANN, Expert Systems, Multiple Discriminant Analysis
3	[30]	165 GCO and 165 non-GCO, 1978-1985, United States	ANN
4	[31]	24 GCO and 25 non-GCO, 1989-1990, United States	Fuzzy Clustering, Expert Systems, M-estimator Discriminant
5	[9]	23 failed (GCO) and 192 healthy, 1986-1988, United States	ANN (backpropagation, categorical learning network, and probabilistic network)
6	[4]	165 GCO and 165 non-GCO, 1980-1987, United States	Logistic Regression (LR), ANN, Decision Trees
7	[5]	271 GCO and 10,047 unqualified opinions, 2002-2004, United States	AntMiner + , C4.5, Support Vector Machine (SVM), LR
8	[32]	73 GCO and 73 non-GCO, 2001-2011, Iran	CART, Naïve Bayes Bayesian Network (NBBN)
9	[2]	55 GCO and 165 unqualified opinions, 2004-2008, Taiwan	Random Forest (RF), Rough Set Theory
10	[11]	48 GCO and 124 non-GCO, 2002-2013, Taiwan	ANN, CART, SVM
11	[12]	49 GCO and 147 non-GCO, 2001-2016, Taiwan	Stepwise Regression, ANN, CART, C5.0
12	[10]	195 GCO and 195 unqualified opinions, 1986-2000, United States	Random Forest (RF)
13	[13]	88 GCO and 264 non-GCO, 2002-2019, Taiwan	Deep Neural Networks (DNN), Recurrent Neural Network (RNN), CART
14	[14]	86 GCO and 172 non-GCO, 2004-2019, Taiwan	Long Short-Term Memory (LSTM), Gated Recurrent Unıt (GRU)
15	[6]	134 GCO and 402 non-GCO, 2000-2019, Taiwan	Artifical Intelligence (AI), CART, CHAID, Extreme gradient boosting (XGB), ANN, SVM, C5.0

Table 1 presents previous studies that use ML methods in the estimation of GCO by distinguishing between GCO and non-GCO companies. For example; [16] compared the predictive power of ANN, expert systems (ES) and multiple discriminant analysis (MDA) models for GCOs and the results of their study show that the artificial neural network model has superior predictive ability. [5] compared the prediction accuracy of AntMiner + , C4.5, SVM and Logistic Regression (LR) techniques for GCO and reveal that the prediction success of SVM and LR models is higher. [2] propose a hybrid model that combines random forest (RF) and rough set theory (RST) approaches to predict GCO. Their results show that the proposed hybrid RF + RST approach has the best classification rate. [11] apply three ML methods such as neural network (NN), classification and regression tree (CART), and support vector machine (SVM) to construct going concern forecasting models using the least absolute shrinkage and selection operator (LASSO) for variable selection. They reveal that the LASSO-SVM model has the highest prediction accuracy (89.79%) in predicting GCO. The results of the study by Chen (2019), in which they adopted a two-stage path, with the first stage being stepwise regression (SR) and artificial neural network (ANN), and the second stage being CART and C5.0 algorithm, indicate that the SR-CART model has the highest accuracy rate (87.42%) in predicting GCOs. [14], using long short-term memory (LSTM) and gated recurrent unit (GRU) to predict GCOs, find that the LSTM model has the highest prediction accuracy (96.15%). The results of the study by [13], in which deep neural networks (DNN), recurrent neural networks (RNN) and CART methods were used, show that the CART-RNN model has the highest prediction accuracy (95.28%) for predicting going concern.

## 3. Materials & methods

This section provides information on the dataset, feature selection methods, and classifiers used in this study. We use several benchmark models such as the support vector machine (SVM), the k-nearest neighbors algorithm (KNN), random forest (RF), Adaptive.

**Fig 1:**
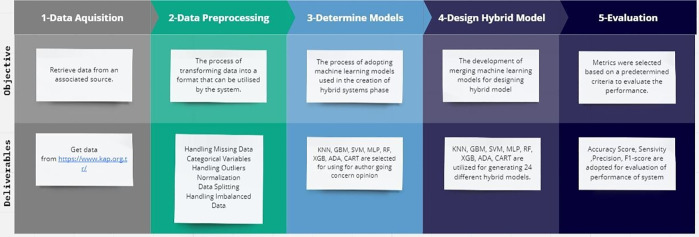
Proposed model diagram.

Boosting (ADA), Classification and Regression Trees (CART), Gradient Boosting Machine (GBM), XGBoost (XGB) to build the optimal hybrid ML model for GCO prediction. A flow diagram of the proposed system with a detailed description of each phase is shown in Fig 1. As illustrated in Fig 1, auditors’ opinions were retrieved from https://www.kap.org.tr/ internet address. In the second part, a check of the data set was carried out. There were no cases of missing data or outliers in the data set. As the difference between classes is small, the problem of unbalanced data sets has not been taken into account. Given that the distribution of classes in the analysed dataset is balanced, the comparative evaluation across classical and hybrid models remains unbiased. While dataset balance itself is a good analytical practice rather than a methodological contribution, we additionally verified that artificial imbalance (70:30 ratio) produced less than a 2% variation in accuracy and F1 metrics. This confirms that model performance improvements derive from algorithmic design rather than sampling effects. In the third part, in the development of hybrid systems, we have chosen to implement a large number of ML methods. A variety of models were developed, including systems based on clustering, regression, and decision trees. In the fourth part, RF, XGB, GBM, MPL, KNN, SVM based models were crossed with Ada, GBM, CART, RF based models. Therefore, a total of 30 ML groups were created, consisting of 24 hybrid systems and 6 basic ML models. In the final part, hybrid and classic models were tested with accuracy, sensitivity, precision, and f1-score.

### 3.1. Dataset

To fulfill the empirical target of this study, our study is based on 234 firm-year data of 98 randomly selected non-financial companies listed in Borsa Istanbul (BIST) for the period 2017–2021. These companies are selected because they have been traded on BIST for many years. Of the 234 companies in the sample, 99 are GCO and 135 are non-GCO companies. As in previous studies in this area, we exclude financial institutions due to special accounting rules [33]. Table 2 shows the sectoral classification of the sampled groups according to Economic Activities. The dataset includes 19 industrial categories in Turkey from 2017 to 2021.

**Table 2:** Classifying the sample according to Economic Activity.

No	Sector	Number of Company
1	Fundamental metal industry	13
2	Education, health, sports and other social services	16
3	Electricity, gas and water	2
4	Real estate activities	1
5	Food, beverage and tobacco	22
6	Administrative and support service activities	4
7	Production	91
8	Construction and public works	11
9	Paper and paper products printing	1
10	Chemicals pharmaceuticals oil rubber and plastic products	11
11	Mining and quarrying	9
12	Metal goods, machinery, electrical devices and transportation vehicles	9
13	Forest products and furniture	1
14	Hotels and restaurants	13
15	Based on stone and soil	2
16	Technology	6
17	Textile, clothing and leather	6
18	Wholesale and retail trade	15
19	Transportation and storage	1

The dataset of our study is based on financial (liquidity, solvency, turnover, and profitability) and non-financial (firm size and type of audit firm) 23 variables that are frequently used in previous studies on GCO estimation. The description of these variables used as estimators of the research model is presented in Table 3.

**Table 3:** The research model predictors.

No	Predictor	Predictor Description
1	L1	Current ratio: Current assets/current liabilities
2	L2	Liquidity ratio (Quick ratio: Quick assets/ current liabilities)
3	L3	Cash ratio: cash & equivalents/current liabilities
4	L4	Cash flow from operations/ total liabilities
5	L5	Net working capital to assets ratio: Net working capital/ total assets
6	S1	Debt ratio: Total debt to total assets (Total liabilities/ total assets)
7	S2	Debt to equity: Total debt to equity (Total liabilities/ equity)
8	S3	Long term debt to total assets (Long term liabilities/ total assets)
9	S4	Long term debt to equity (Long term liabilities/equity)
10	S5	Financial leverage: Total assets/ equity
11	S6	Proprietary ratio: Equity/ total assets
12	T1	Inventory turnover ratio: Cost of goods sold/average inventory
13	T2	Fixed asset turnover: Net sales/ (Gross fixed assets – accumulated depreciation)
14	T3	Asset turnover ratio: Net sales/ average total assets
15	T4	Current assets turnover: Net sales/current assets
16	T5	Working capital turnover: Net sales/ (current assets-current liabilities)
17	T6	Equity turnover ratio: Net sales/ average shareholders’ equity)
18	P1	Net profit ratio: Profit after tax/ Net sales
19	P2	Return on assets (ROA) (Profit after tax/ total assets)
20	P3	Return on equity (ROE) (Profit after tax/ equity)
21	P4	Retained earnings/ total assets
22	SIZE	Log of total assets
23	BIG4	Type of auditor

### 3.2. Benchmarking models

#### 3.2.1. Classification and regression tree (CART).

The CART algorithm is an algorithm based on decision trees that can be utilised for both classification and regression problems [34]. CART aims to simplify decision structures in complex data sets. The algorithm divides heterogeneous data sets into homogeneous subgroups based on a specified target variable. It recursively divides the training data into smaller subsets

using binary splits. The partitioning of data into two subsets, then repeat the process to determine the next condition of partitioning in each subset.

The CART methodology consists of three steps: (1) constructing the maximum tree; (2) selecting the optimal tree size; and (3) classifying or generating new data based on the constructed tree.

The derivation of decision rules is one of the key aspects of building CARTs. In this context, decision rules are determined using Gini rules. Gini (G) can be calculated, as expressed in Equation (1).


$$G=1-\sum_{i=1}^{n}p_{1^{2}}$$ (1)

where n represents the number of classes, the probability of a randomly chosen element in the node being labeled as class i is p_i_.

The Gini index can be calculated using the following equation when data D are classified into *D*_1_ and *D*_2_ by a given variable x, on the basis of a given characteristic f:


$$G(D,x)=\left(\frac{D_{1}}{D}\right)*G\left(D_{1}\right)+\left(\frac{D_{2}}{D}\right)*G\left(D_{2}\right)$$ (2)

#### 3.2.2. Random forest (RF).

The RF algorithm is suggested by [35]. Random Forest (RF) allows building different models and creating classifications by training each decision tree on a different sample of observations using multiple decision trees. After several trees have been constructed, the best attributes are selected using the random subset of attributes [36]. RF is flexible and easy to use, as it can be applied to both classification and regression problems. The steps are as follows.

- The bagging sampling method is used to generate K training sets from the original training set M, with each set containing N samples.
- Train K training sets to generate K CART decision tree models
- For the features of a single decision tree model, the optimal split attribute of the current node is selected based on the GINI index to generate branch nodes, resulting in the creation of a single decision tree.
- A random forest was formed from the K decision trees generated.

In Equation (1) class and the probability identify the Gini of each of the branches at a node to decide which of the branches is more likely to be seen. Entropy governs the branching of nodes in a decision tree. Entropy is calculated by using the following function in Equation (3).


$$\text{Entropy}=-\sum_{i=1}^{n}\;p_{i}*\log_{2}\left(p_{i}\right)$$ (3)

where ${\;p}_{i}$ denotes the relative frequency of the class being considered in the dataset, and n denotes the number of classes. The probability of an outcome is used to determine how the node should be branched using entropy.

#### 3.2.3. K-nearest neighbors (KNN).

The k-nearest neighbour classifier is based on the distance metric. Minkowski and Euclidean distance metric to determine label similarity, which is the most widely used and most efficient metric for this purpose. The k-nearest neighbour (kNN) algorithm aims to identify the k-nearest neighbours of the query from the dataset and allocate a class label to the neighbourhood by label to the neighbourhood using the majority decision rule.

Suppose that in a D-dimensional space Dataset S = ${⋃}_{i=\text{1}}^{N}\{(y_{i},x_{i}\}$ represents a training set with N instances from T classes. The variable $c_{i}$ denotes the class label for $y_{i}$. $c_{i\;}\in \left\{t_{i\;},t_{\text{2}\;},..t_{T}\right\}$ [37]. The distance between the query point and the other data points must be calculated to identify which data points are closest to a given query point. Distance metrics are used to create decision boundaries that divide query points into different regions. The Euclidean distances between the given query x and each of the training instances in S are computed as follows:


$$\begin{array}{c} d(x,y)=\sqrt[{\;}]{\;\sum {\mathrm{\backslash nolimits}}_{i=\text{1}}^{n}{\left(y_{i}-x_{i}\right)}^{\text{2}}} \end{array}$$ (4)

Deciding can be expressed as follows:


$$D_{i}(x)=\;x_{i},\;i=\text{1},\text{2},\text{3},..,T$$ (5)

The decision rule according to Equation (5) is: if $D_{i}(x)={max}_{i}x_{i}$ then x $\in \;t_{i\;}\;$ on D-dimensional space $x_{i}|t_{i\;}$ for i = 1,2,..,T (probability distributions) $\;P_{i}$. Order the training dataset pairs of ($x_{\text{1}}-$x), ($x_{\text{2}}-$x), …., ($x_{n}-$x)

#### 3.2.4. Gradient-Boosting classifier (GBM).

[38] presented the gradient boosting model, which is an ensemble model of machine learning. To improve the accuracy and robustness of the final model, the model combines several weak learners. The gradient boosting model begins by creating a single leaf and constructing regression trees. Assuming that Dataset D = ${⋃}_{i=\text{1}}^{N}\{(y_{i},x_{i}\}$, the objective of gradient boosting is to obtain an approximation. A regression tree is built using an iterative process of splitting data into nodes or branches, creating smaller groups. At the beginning, all instances are placed in the same group. The data is divided into two sub-sets by testing every available predictor for every possible split [39]. The predictor aims to minimize the residual error of a given loss function and the next predictor goes on to create more trees using this method until it is no longer possible to improve the desired threshold or fit.

The Loss Function (L) and the Gradient Boosting Approximation Function (G) are referred to L(y,F(x)) and G(x), respectively.

Initally, a constant approximation of G(xis determined as:


$$\begin{array}{c} F_{\text{0}}(x)=\;\frac{\text{1}}{N}\sum {\mathrm{\backslash nolimits}}_{i=\text{1}}^{N}y_{i} \end{array}$$ (6)

The initial prediction $F_{\text{0}}(x$ is typically chosen to average all target labels y. This initial prediction indicates best estimate without any feature input (X) [40]. Minimize the residual error of the given loss function:


$$r_{it}=-\left(\frac{\partial L\left(y_{i},F_{t-\text{1}}\left(x_{i}\right)\right)}{\partial LF_{t-\text{1}}\left(x_{i}\right)}\right)for\;i=\text{1},\ldots .N$$ (7)

Where t is the estimator count, with loss function calculate the difference between $y_{i}$ and $F_{t-\text{1}}\left(x_{i}\right)$.

In Equation (8). Fit a regression tree:


$$e_{t}\left(x\right)=argmin_{t}\sum_{i=1}^{N}{\left(r_{it}-e\left(x_{i}\right)\right)}^{2}$$ (8)

$$F_{m}(x)=F_{m-\text{1}}(x)+\;\mu *e_{t}(x)$$ (9)

where n represents the learning rate, a value between 0 and 1 that determines the weight of each weak model. In Equation (8), a regression tree is fitted to the current residuals in order to update the model.

#### 3.2.5. Support vector machine.

The Support Vector Machine (SVM) is a type of neural network model that can determine the optimal solution and reduce model complexity while maintaining learning performance [41]. The Support Vector Machine (SVM) was initially developed for binary classification. However, it can also be applied to multi-class classification problems by combining multiple binary classifiers [42]. Support Vector Machines (SVM) offer important advantages in processing high, small samples and non-linear data, and exhibit high robustness and generalisation performance in solving regression and classification problems [43]. Assuming that in a D-dimensional space and let assume that there is a classification problem Dataset D = ${⋃}_{i=\text{1}}^{N}\{(y_{i},x_{i}\}$, where $x_{i}$ is input and $y_{i}$ is output and ∈ {$c_{\text{1}}$,…,$\;c_{t}$}. The objective function has been modified to enable multiclass classification to be performed simultaneously and is given by:


$$min_{w,b,e}\left[\frac{1}{2}\sum_{i=1}^{t}||w|{|}^{2}+C\sum_{i=1}^{k}\sum_{r\neq y_{i}}e_{i}^{r}\right]\;for\;i=1,....,k$$ (10)

under these conditions.

${w_{y_{i}}.x}_{i}+b_{y_{i}}\geq {w_{r}.x}_{i}+b_{r}+\text{2}-e_{i}^{r}\;$and $\;e_{i}^{r}\;\geq \text{0}$

where $y_{i}\;$∈ {$c_{\text{1}}$,…,$\;c_{t}$} and r ∈ {1,…, t}/$y_{i}$

#### 3.2.6. Multilayer perceptron (MLP).

**Fig 2:**
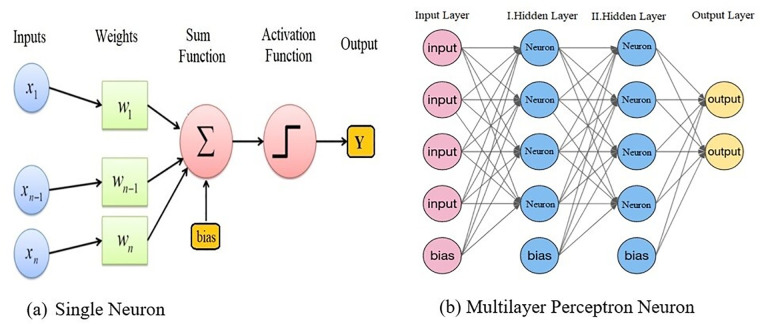
Multilayer Perceptron Diagram.


MLP is one of the most widely used feed-forward back-propagation artificial neural networks. The MLP is made up of a minimum of three node layers. The Input layer, Hidden layer, and Output layer (Fig 2). Neurons are the basic units that compose each layer. A neuron consists of a sum function and an activation function. Fig 2 (a) illustrates the function τ(x) and the operational principle of the neuron with confidence. Input layer variables are multiplied by their corresponding weight coefficients and then accumulated by the sum junction. The output value can be obtained from the combined summation and bias of the summation and bias using the activation function. The neuron’s operating principle can be defined through the following Equation (11) [28].

$$y_{i}^{k}=\tau (\sum_{i=1}^{n}w_{i}^{k}x_{i}+b_{i}^{k}$$ (11)

where order of the neuron is denoted by ‘i’ and order of layer presents by ‘k’. Hence, the value at the ith neuron in the kth layer is expressed by the term $y_{i}^{k}$. The weight at the ith neuron in the kth layer is given by the term $w_{i}^{k}$.


$$h_{i}^{\left(1\right)}=\tau^{1}(\sum_{i=1}^{n}\;w_{i}^{1}x_{i}+b_{i}^{1})$$

$$\begin{array}{c} h_{i}^{\left(k\right)}=\;\tau^{k}(\sum {\mathrm{\backslash nolimits}}_{i=\text{1}}^{n}w_{i}^{k}h_{i}^{k-\text{1}}+b_{i}^{k}) \end{array}$$

$$\begin{array}{c} y=\;\tau^{k}(\sum {\mathrm{\backslash nolimits}}_{i=\text{1}}^{n}w_{i}^{k}h_{i}^{\left(k-\text{1}\right)}+b_{i}^{k}) \end{array}$$ (12)

Fig. 2 (b) extends this concept to a multilayer network in which an input layer transmits information to two hidden layers through fully connected weighted links. Each hidden layer consists of several neurons that apply non-linear transformations to learn intermediate feature representations. The final output layer generates prediction values based on the learned representations. The arrows in the figure indicate the direction of forward information flow, while the presence of bias units ensures the model has the flexibility to shift activation thresholds. Together, these components enable the MLP to learn complex, non-linear relationships within the financial dataset used in this study.

#### 3.2.7. Adaptive boosting (AdaBoost).

**Fig 3:**
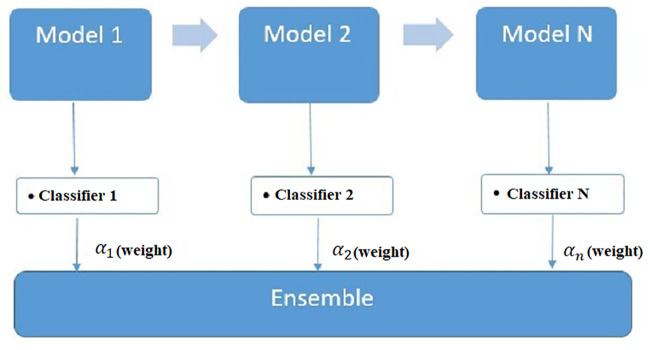
Adaptive boosting diagram.

Adaptive boosting (AdaBoost) is an ensemble ML algorithm developed by [44]. It is used for the solution of problems in classification and regression. Recently, the AdaBoost model has gained popularity in various fields [45–47]. AdaBoost merges multiple weak classifiers iteratively to generate a unique strong classifier. The extent to which a weak classifier is inaccurately trained is determined by the weight assigned to each training sample [48,49]. Fig. 3 shows that ensemble learning is based on several weak classifiers by changing the sample weights of the dataset several times throughout the learning process. Dataset D = ${⋃}_{i=\text{1}}^{N}\{(x_{i}{,y}_{i}\}$, where $x_{i}$ is input and $y_{i}$ is output vectors, N represents the number of samples. Each classifier(N) contributes to the final decision via a weighted combination, where $\alpha_{\text{1}},\alpha_{\text{2}},\ldots ,\alpha_{N}$ represent non-negative scalar weights associated with each classifier. The weights indicate the relative importance or the predictive confidence of each model within the ensemble, and these may be determined based on such metrics as validation accuracy, optimization criteria or algorithm-specific learning dynamics. The final prediction is computed as a weighted aggregation of all classifier outputs, allowing the ensemble to leverage model diversity and reduce variance, bias, or overfitting effects that may arise from individual classifiers.

First, weights are assigned to data points, and initially, all weights are equal. Where N is the total number of data points in the data set. The first weak classifier, model 1, is obtained after appropriate training. According to the classification result of the previous weak classifier, the sample weights are adjusted.

A decision node is created for each of the features and then the Gini index of each tree is computed. The tree with the lowest Gini index is the first node as expressed in Equation (1).

The weak learner *l*_*i*_ builds from the training dataset using w, which minimizes the weighted error. The weight represents the confidence ($k_{j}$) of the jth model.

Weak classifiers can be replaced with stronger ones, thanks to AdaBoost.


$$e_{j}=\sum_{i:l_{j}\left(x_{i}\right)\neq y_{i}}w_{i}^{j}$$ (14)

$$k_{j}=\frac{\text{1}}{N}{log}_{e}(\frac{\text{1}-e_{j}}{e_{j}})$$ (15)

$$w_{i}^{j+\text{1}}=e^{-y_{i}l_{i}{(x}_{i})k_{j}}w_{i}^{j}$$ (16)

$$\begin{array}{c} H(x)=\sum {\mathrm{\backslash nolimits}}_{j=\text{1}}^{D}k_{j}h_{j}(x) \end{array}$$ (17)

#### 3.2.8. XGBOOST.

XGBoost is an improved ML algorithm that extends the gradient-boosting decision tree algorithm. It is proficient in building boosted trees highly and is used to facilitate parallel computing. XGBoost is initially proposed by [50]. The algorithm’s key features are that it achieves high predictive power, prevents overfitting, quickly manages empty data, and effectively handles datasets with missing values. According to [50], XGBoost operates ten times faster than other popular algorithms. The main goal of XGBoost is to increase prediction accuracy by utilising the learning from previous weak learners and implementation of new weak learners, specifically designed to address and correct the remaining errors [51]. The purpose of the XGBoost algorithm is the optimisation of the target’s function.


$$\begin{array}{c} Target(x)=\sum {\mathrm{\backslash nolimits}}_{i=\text{1}}^{n}l(y_{i},\hat{y_{i}})+\sum {\mathrm{\backslash nolimits}}_{j=\text{1}}^{T}\text{⌽}(f_{j}) \end{array}$$ (18)

where l represents the loss function that calculates the difference between the true value $y_{i}$ and the predicted value $\hat{y_{i}}\;$ and n represents the dimension of the feature vector. The symbol $\begin{array}{c} \text{⌽} \end{array}$ represents the regularization term that is added to the target function. The target function is to handle the complexity of the model and avoid overfitting. $f_{j}$ is the function of the jth tree. $\hat{y_{i}}$ is written with the Equation (18). as follows.


$$\begin{array}{c} \hat{y_{i}}=\sum {\mathrm{\backslash nolimits}}_{j=\text{1}}^{T}f_{j}\left(x_{i}\right)=\;{\hat{y_{i}}}^{k-\text{1}}+f_{j}\left(x_{i}\right) \end{array}$$ (19)

$\left(f_{j}\right)$ is the normalisation term used to describe the complexity of the tree structure.


$$\left(f_{j}\right)=\beta T+\frac{1}{N}\alpha \sum_{j=1}^{T}w_{j}^{2}$$ (20)

where T is the number of leaf nodes in the tree, β determines the minimum descent value of the loss function required for node splitting, and α represents the L2 regularisation term performed on the weights.

### 3.3. K-fold Cross-validation

**Fig 4:**
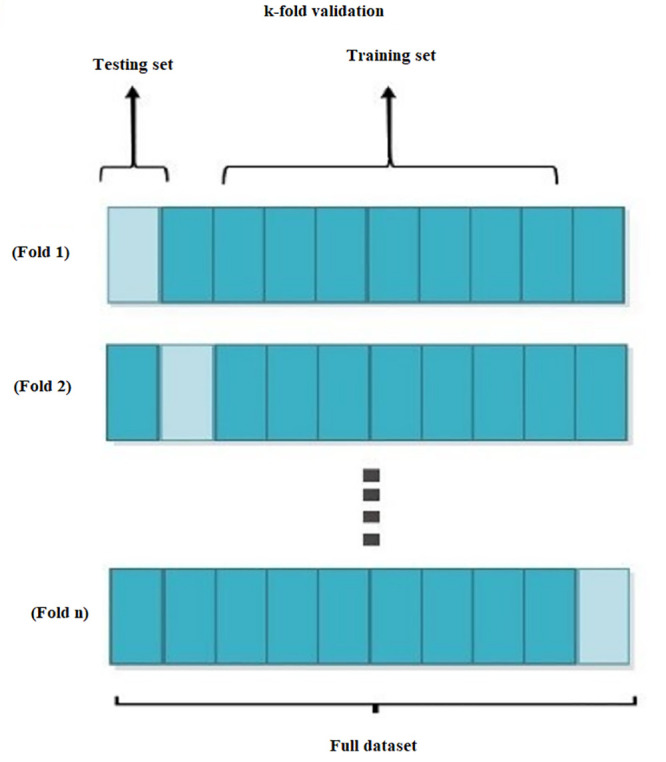
K-fold cross validation diagram.

In the use of ML for practical applications, over-fitting is a common problem issue. The issue of overfitting can be addressed through the use of cross-validation [52]. The model weights for the averaging prediction were determined using the fold cross-validation criterion. The model weights were chosen by minimising the sum of the squares of the prediction errors from all groups [53]. The k-fold cross-validation involves dividing the sample into equally sized K subsets and using each group as a validation sample to assess the model’s performance. Of this subset, K-1 is used to train the models and the rest is used to test them. When each unique subset has been validated once, the operation is repeated k times [15]. It is possible to get the accuracy of the K models, and the performance of the K-CV classifier model is assessed according to the average accuracy of the K models. The overall evaluation metrics of the models are computed k times, from which different ML metrics (Accuracy Score, Sensivity, Precision, F1-Score) can be calculated. Fig. 4. shows the k-fold cross-validation diagram.

The most frequent settings of K are 5 and 10, and this corresponds to perform a leave-one-out cross-validation when K = n [53]. In this study, k is set at 10, as this is thought of as giving an unbiased estimation of the error rate of the test [54].

### 3.4. Hyper Tuning parameters

Hyper-parameters tuning is a significant process to determine the optimal machine learning parameters. Determining the best hyperparameters is a laborious process and takes a long time, especially when the objective functions are difficult to ascertain, or a substantial number of parameters are required to be tuned. In this study, RandomizedSearchCV is utilized for optimizing the hyperparameters that explore enabling efficiently the search space via stochastic sampling combined with k-fold cross-validation. In the Table 1A, determining the best hyperparameters were demonstrated in appendix section.

### 3.5. Designing hybrid systems

The initial settings are very important for the results of the ML model. The optimisation algorithms are employed to adjust the ML parameters. In our hybrid approach RF, XGB, GBM, MPL, KNN, and SVM are utilized for predicting GCO. The Adaptive Boosting (Ada), Gradient.

**Fig 5:**
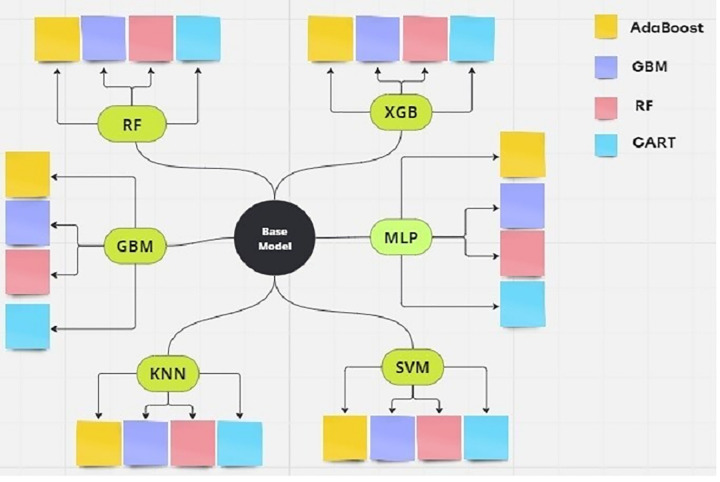
Hybrid model diagram.

Boosting Machine (GBM), Random Forrest (RF), Classification and Regression Trees (CART) were used to train the RF, XGB, GBM, MPL, KNN, SVM models. Hence, a total of 24 hybrid systems were generated. Fig 5 displays the details of the hybrid system network. As illustrated in Fig 5, the hybrid model construction process that was utilised in the present study is depicted. Initially, six classical machine learning models (i.e., RF, GBM, XGB, MLP, KNN, and SVM) are trained as base learners, as illustrated at the centre of the diagram. Subsequently, each base learner is combined with one of four ensemble strategies—AdaBoost (yellow), GBM (blue), RF (red), or CART (cyan)—to generate hybrid variants. The colour-coded blocks represent the feature-selection or boosting component applied to each base model, resulting in 24 hybrid configurations in addition to the six original base models.

## 4. Result and discussions

### 4.1. Evaluation metrics

To evaluate the study’s performance, we constructed a confusion matrix consisting of true positives (tp), false positives (fp), true negatives (tn), and false negatives (fn). The most commonly used metric for performance assessment is the accuracy score. In this study, our model performs binary class classification, so the assessment metrics consider two classes, and the equation is as follows:


$$Accuracy\;Score=\;\frac{TP+TN}{TP+FP+FN+TN}$$ (21)

$$Sensivity\;=\;\frac{TP}{TP+FN}$$ (22)

$$Precision\;=\;\frac{TP}{TP+FP}$$ (23)

$$\text{F1}-\text{score }=\text{2}*\;\frac{\;(Sensivity*Precision)}{\left(Sensivity+Precision\right)}\;$$ (24)

### 4.2. Comparison between classical and hybrid models

This phase describes the details of the hybrid and classical ML models used in this study. As mentioned, we are dealing with a binary classification problem, using a set of predictor features as inputs. The hybrid and classical models aim to categorise the firms in the dataset as GCO or non-GCO. The results of the classical benchmark models are evaluated using all 23 variables. The evaluation of model performance using acc_score will not be complicated since there is no imbalance in the dataset. As shown in Table 4, balanced data distribution prevented distortion of evaluation metrics such as accuracy and F1-score, allowing the hybrid models’ gains to be attributed to algorithmic structure rather than class imbalance correction.The performance of six classical machine learning models was assessed by looking at Table 4, which denotes the performance values measured on test data using 10-fold cross-validation. The Table 4 shows that RF provides the most accurate predictions and the lowest accuracy score was received by SVM among classical ML models.

**Table 4:** Performance evaluation of basic classical models (%).

No	Model	Acc	Precision	Recall	F1-Score
1	XGB	93.57	94.84	89.79	92.14
2	RF	91.45	88.64	91.95	90.19
3	SVM	88.45	83.45	91.89	87.27
4	GBM	88.45	86.44	86.89	86.50
5	MPL	86.24	84.61	84.74	84.31
6	KNN	76.45	75.44	68.33	70.24

The primary objective of his study is to develop appropriate hybrid systems. Table 5 presents the parameters adopted by the models to create hybrid systems. This study uses RF, AdaBoost, GBM, and CART, as models to choose important variables, remove noise, and boost model accuracy. Table 5 reveals that the model suggesting the most variables is RF and the model suggesting the fewest variables is GBM.

**Table 5:** The list of variables selected by models.

No	Model	Variables
1	RF	L1, L2, L3, L5, S6, T5, P1, P2, P4
2	AdaBoost	L2, L4, S4, T1, T5, P4, SIZE
3	GBM	L2, L3, T5, P4
4	CART	L1, L2, T5, P4, SIZE, BIG4

**Fig 6:**
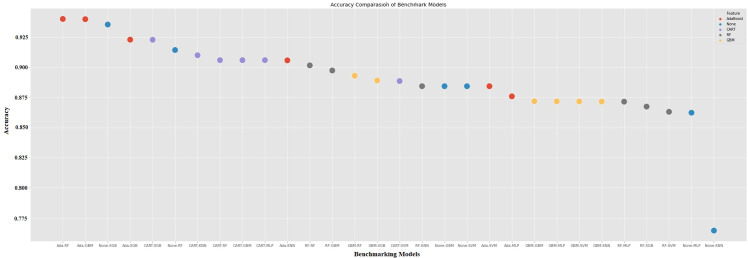
Performance evaluation of all models.

In this section, the success of the models with the inputs provided by the ML is evaluated. Thus, It has been determined which hybrid model is the best. Fig 6 displays the feature selection models that are combined with the base classical models. The merging of the AdaBoost models with the basic models is indicated by the red dots, blue dots denote where None-feature seleciton has merged with the base, CART that has merged with the base models is indicated by purple dots, grey dots indicate RF base models, and yellow dots indicate GBM which have merged with the base models in Fig 6. Fig 6 demonstrates that the first five hybrid models achieved higher accuracy scores than the base model, which had the highest accuracy score among the basic models. Again, Fig 6 shows that the last five hybrid models get lower accuracy scores than the base model, which had the lowest accuracy score among the basic models. The choice of a hybrid model does not always lead to better results. As illustrated in Table A1 (in the appendix) and Fig 6., certain traditional models such as XGB and RF achieved higher accuracy than some hybrid variants (e.g., SVM-Ada, SVM-GBM). These experimental results illustrate that performance improvements are not guaranteed as a result of hybridisation. This aligns with prior research reporting that hybrid models may introduce redundancy or overfitting when model components are poorly matched [2,4]. While hybrid models are regularly developed to improve predictive precision, prior research and our trial outcomes (see Table A1 and Fig 6) show that their superiority is dependent on the circumstances.

Examining the initial five models that generate the most accurate prediction accuracy score in Fig 6, we observe that the feature selection algorithms used include ADA and CART. It was noted that six variables were used by CART and seven by RF. The hybrid model FR-SVM, which received the lowest prediction score among the hybrid models, made predictions using four variables.

**Fig 7:**
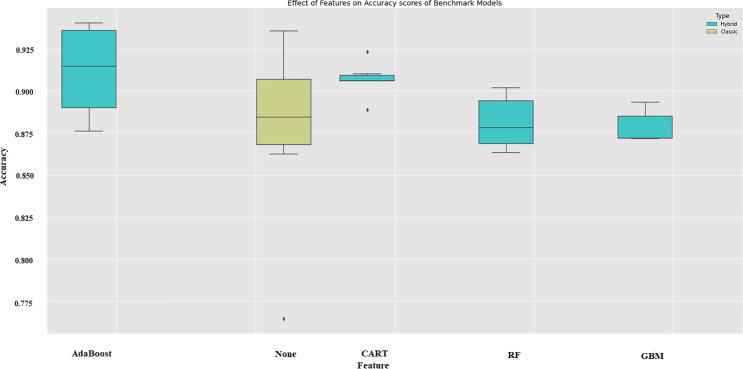
Effect of feature selection on accuracy score among benchmark models.

Fig 7 shows the effect of the most effective parameters on hybrid systems after they have been determined using ML models. Looking at Fig 7, it is obvious that without features selection the results are highly volatile. It is clear that the features that are selected by the cart model are the more stable ones. The predictive ability of the basic models is similar when using features, as adopted by the cart model. The use of AdaBoost in hybrid models can result in extreme prediction values, as illustrated by the points in Fig 7. Fig 7 shows the variability of accuracy scores across feature selection strategies used in the hybrid benchmark models. Error bars shown in box plots represent the distribution of model accuracies from repeated cross-validation folds. Wider error bars indicate higher variability and hence less stable performance across folds, on the otherhand narrower error bars reflect more consistent prediction behavior. As seen in the AdaBoost-based hybrids, the error bars are significantly wider, indicating that the features selected by AdaBoost exhibit higher volatility, causing accuracy values to fluctuate more significantly across folds. This instability suggests that AdaBoost-driven feature weighting is sensitive to sampling differences, conducting to less generalizable results. Conversely, the use of CART-based feature selection shows much more precise error bars, pointing out that CART produces more consistent and reproducible feature subsets. The RF and GBM feature selectors fall between these two extremes. While both provide occasional peaks of high accuracy, their wider interquartile ranges indicate that performance can vary significantly depending on the training layer. The error bar patterns demonstrated in Fig 7 show that CART generated hybrid configurations with the highest consistency. On the otherhand, AdaBoost, while occasionally producing fairly accurate results, tends to introduce more variance.

One of the most important metrics is recall (sensitivity), which explains how many of the predictions we made were positive. In cases where the cost of a false negative is high, the sensitivity score is also a metric that will assist us. It should be as high as it can be. In this study, the information generated for the auditor’s report. If the model marks an accepted GCO as non-GCO, the consequences of a situation pose a problem for a company. Hybrid models, which are Ada-RF, CART-XGB, None-RF, and Ada-GBM, have 94.00%, 92.94%, 91.94%, and 91.94% the highest predicted precision scores, respectively. None-KNN got the lowest recall value, 68.33%.

**Fig 8:**
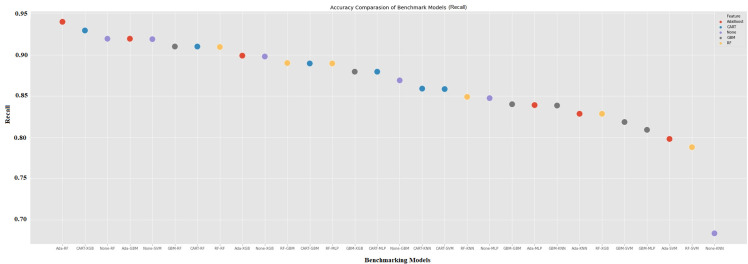
Recall comparasion of benchmark and hybrid machine learning models.

**Fig 9:**
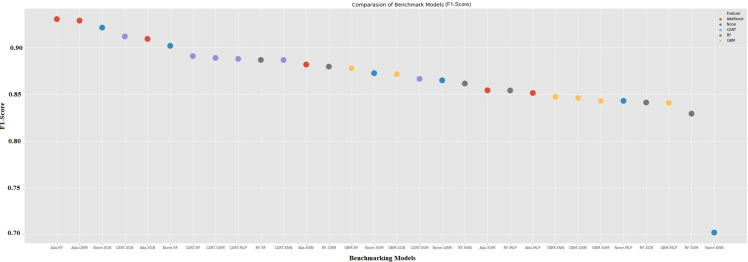
F1-score comparasion of benchmark and hybrid machine learning models.

Fig 8 and 9 show the net performance patterns across the 30 benchmarks and the hybrid model configuration. The reviewer expressed concern about false negatives, which lead to significant audit costs. The emphasis is on recall and F1-score metrics. Accuracy is less important. These figures show that models with good recall performance also tend to have high F1-scores, making them better suited for continuous business forecasting. The main aim of continuous business forecasting is to prevent financially distressed companies from being incorrectly classified as healthy.

In both models, the top rankings for recall and F1-score are consistently held by AdaBoost-based hybrid models (e.g., Ada–RF, Ada–GBM, Ada–XGB).These models outperform most of the baseline non-hybrid models, achieving recall values above 0.93 and F1-scores above 0.92. This model reflects AdaBoost’s ability to iteratively reweight misclassified observations, which appears particularly advantageous for capturing early signals of distress. Because distressed firms are generally more difficult to classify, AdaBoost’s focus on misclassified samples is likely to make it more sensitive.

When considering accuracy alone, no discernible performance difference emerges. This is also seen in the lower-ranked models in Fig 8 and 9. These models demonstrate relatively high accuracy thanks to the balanced nature of the dataset. However, recall values fall below 0.82, indicating a higher risk of false negatives. This distinction is critical for audit practitioners and regulators because a model with slightly lower accuracy but higher recall is more reliable in identifying firms requiring further investigation. This means both figures highlight the importance of using recall and F1-score as key evaluation metrics in the context of ongoing business.

According to Fig 8 and 9, the results show that hybrid boosting and hybrid tree-assisted models, particularly AdaBoost- and CART-based combinations, provide the strongest performance in identifying distressed firms. These experimantal results demonstrate the suitability of ensemble-assisted hybrid approaches for audit risk applications. In these cases, avoiding false negatives is more important than maximizing overall classification accuracy.

The harmonic average of the precision and recall values is another important metric, the F1 score. The reason to use harmonic averaging rather than simple averaging is to avoid ignoring extreme cases. The main reason for the use of the F1 Score value instead of the Accuracy value is to avoid incorrect model selection in unbalanced data sets. The 5 models with the highest F1 scores are Ada-RF, Ada-GBM, None-XGB, CART-XGB, and Ada-XGB, 93.04%, 92.88%, 92.14%, 91.19%, and 90.93, respectively. Again, the hybrid model RF-Ada had the highest f1.

**Fig 10:**
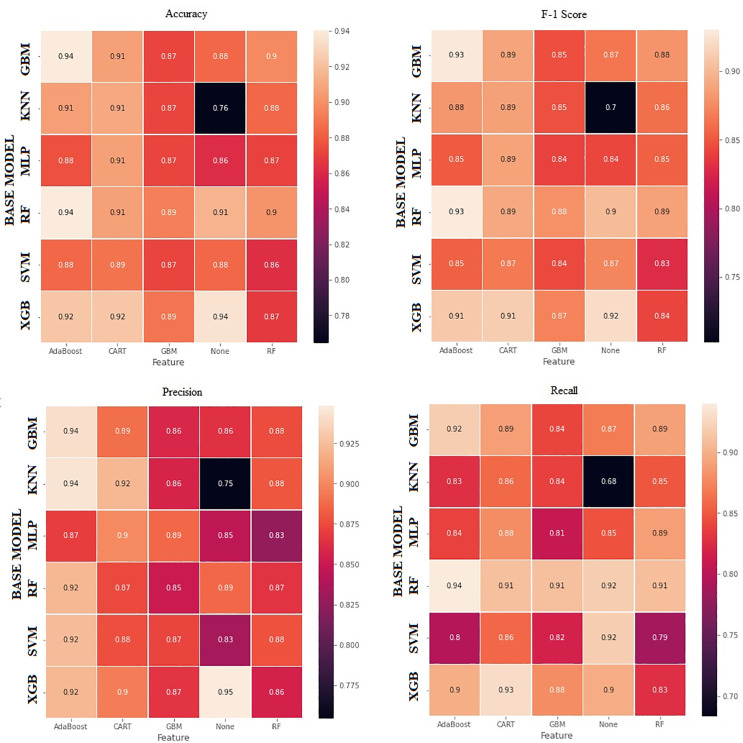
Performance heatmaps for benchmark and hybrid models across four metrics.

The actual and predicted values in a classification problem are shown in the confusion matrix table. The Fig 6 above was made using a confusion matrix table. The key details of the study are contained in Fig 10. The most used metrics for classifying ML problems have been evaluated. In this text, it focused on the evaluation indices: accuracy, precision, recall, and F-measure.

As we mentioned above, the five models with the highest validation scores are hybrid models. Accuracy is a metric that is commonly used to evaluate the performance of a model, but it is not a sufficient measure on its own, especially with unbalanced data sets that are not evenly distributed. Although the data set used was a balanced one, the success of the hybrid models created has also been evaluated based on other metrics. The precision value is very important, especially when the cost of a false positive is high. Here, if the prediction model marks the companies that should be approved as a rejection instead of acceptance, the company will fail the audit. High precision is an important criterion for us when selecting a model in this case. Hybrid models, which are None-XGB, Ada-KNN, Ada-GBM, Ada-SVM, and CART-KNN, have 94.84%, 94.44%, 94.22%, 92.49%, and 92.22% the highest top 5 predicted precision scores, respectively. It is therefore reasonable to assume that the hybrid model predictions are superior to the other classical methods considered. Hybrid models, which are None-KNN, RF-MLP, None-SVM, None-MLP, and GBM-RF, have 75.44%, 82.71%, 83.45%, 84.61%, and 85.12% the lowest predicted precision scores, respectively. This means that the predictive value of hybrid models can be increased or decreased.

The execution of standard ML algorithms was go along with a benchmarking process that was identify by the data structure and domain context of bankruptcy and audit risk prediction. In this setting, hybrid models, especially those associating AdaBoost and CART techniques, yield the most reliable and comprehensive predictive performance for ongoing business evaluations, as demonstrated by the aggregated results for accuracy, recall, and F1 score. Both Ada-RF and Ada-GBM reveal the highest accuracy while maintaining strong recall and F1 values. This experimental result proposes that boosting rises both the overall predictive power of the model and its ability to detect distressed firms. Tree-based models such as None–XGB and None–RF perform well, but hybrid SVM, KNN, and some combinations of MLP are less successful, have lower accuracy and precision, and have difficulty handling noisy or heterogeneous financial patterns. Overall, these experimentalresults demonstrate that auditors should be cautious when interpreting outputs from margin- or distance-based classifiers and place greater emphasis on signals detected by augmented and tree-structured models, especially those indicating liquidity deterioration, enhanced leverage, and weak cash flow. Hybrid tree enhancement configurations are valuable analytical tools in audit planning, particularly when mitigating the risk of false negative audits by identifying financial distress at an early stage is crucial. Their superior performance supports their use.

The purpose of including multiple models in this study is not only to compare metrics but also to develop a methodological framework and improve interpretation. A variety of hybrid and classical algorithms are designed to test when and why hybridization improves or reduces performance in ongoing business forecasting. When we interpret the experimantal results, ensemble-based combinations such as RF–Ada, RF–RF yield consistent gains because they combine variance reduction (bagging) with bias correction (boosting) and they grap non- linear interactions among liquidity, solvency, and turnover ratios. On the otherhand, SVM-based hybrids frequently underperform due to kernel redundancy and sensitivity to scaling, which demonstrates that hybridization does not always guarantee better performance.

These observations contribute valuable insights for auditors and financial-prediction researcher: prediction framework depends on the suitable learner and feature-selection model rather than on algorithm count. Therefore, this comparisons explains the mechanistic origins of performance differences and offers a reproducible framework for assessing hybrid ML systems in other financial-auditing contexts.

The results go beyond just doing experiments by explaining why different models work better. For example, models that combine feature reweighting and ensemble aggregation can capture the multidimensional structure of financial distress indicators, but distance-based or purely linear classifiers are limited in their ability to detect non-linear interdependencies. This domain-based framework prioritizes the contribution to interpretability rather than model comparison. This contributes to both auditing theory and ML model design.

## 5. Conclusion

In this study, the efficacy of classical and hybrid machine learning models in predicting auditors’ going concern opinions (GCO) was examined using firm-level financial indicators of companies traded on Borsa Istanbul. To estimate the auditor’s GCO, we analyse a total of 30 hybrid and classical ML models based on data from a sample of companies listed on the BIST for the period 2017–2021. Of the 30 models built to predict the auditor’s GCO, the highest and lowest predictive accuracy scores belong to hybrid models. This means that the hybrid models constructed should be studied in detail. Our findings show that hybrid architectures (especially the RF-AdaBoost combination) consistently outperform traditional classifiers and that integrating ensemble mechanisms with nonlinear learners increases predictive capacity in audit-related risk assessment. The results reveal that liquidity, turnover, and retained earnings-based indicators are the most influential determinants of GCO estimates across model families, highlighting their importance in capturing early signals of financial distress.

Beyond empirically ranking model performance, the study provides a structured framework to evaluate when and why hybridization improves prediction accuracy.Comparative analysis demonstrates that performance gains arise not only from combining algorithms but also from the complementary strengths of specific ensemble-learner pairings. This information can guide auditors, regulators, and practitioners seeking data-driven tools to support GCO assessments while reducing subjectivity and audit reporting failures.

Our research provides a robust and reliable forecasting model. If the system of equations is non-linear, it is very crucial to choose ML models to solve it. It is also important to ensure that the data set used to create an accurate prediction model is balanced. We also use k-fold cross-validation for efficient use of the dataset. These are all issues that need to be considered to produce a robust and reliable forecasting model, and none of them have been ignored in this study. In the studies in this field, there is no study in which all these factors are taken into consideration at the same time. In addition, our findings may help auditors in reducing audit reporting failures. The hybrid ML model can also be used to perform a risk assessment of whether the auditor’s new client has uncertainty about going concern. Furthermore, the findings of this study may guide the decisions of financial information users in assessing the uncertainties related to going concern.

Future research should incorporate temporally ordered, floating-origin validation schemes to avoid look-ahead bias and better reflect real-world estimation conditions. To increase the generalizability of the model, it is recommended to expand the dataset to include additional sectors and international markets. Integrating textual descriptions or audit report narratives would also be beneficial. Finally, testing hybrid models in year-end forecasting tasks would be a prudent next step. In financial reporting environments, demand for timely, evidence-based assessments is increasing. Hybrid machine learning (ML) systems have the potential to enhance audit quality and improve early detection of ongoing business uncertainty.

## 6. Limitations and future work

This study has some limitations. First, due to data access restrictions, we are unable to follow such trends, although similar studies in developed countries are likely to have larger samples including more years. On the other hand, banks and financial institutions, investment companies, financial intermediation, and holding companies have been excluded due to their special accounting rules. The last limitation of the study is the varying business and cultural characteristics of Turkey in comparison with other countries.

However, despite the importance of this discovery, there are inherent restrictions when carrying out this kind of study. The current dataset includes only firm-level financial records from Turkey due to data accessibility and confidentiality constraints. Comparable going-concern datasets from other European countries or China are not publicly available in standardized format. While this restricts cross-country generalization, it provides a controlled setting to test model behavior under consistent accounting and regulatory environments. Future studies can extend this framework using international datasets to validate the observed patterns across different institutional contexts.

Although the present study employs 10-fold cross-validation to ensure efficient use of the limited dataset, this evaluation strategy does not explicitly preserve the temporal ordering of observations. Because financial statements inherently follow a chronological structure, randomly shuffling firm-year data may introduce a form of look-ahead bias in which information from later years (e.g., 2020–2021) influences predictions for earlier years (e.g., 2017–2018). This does not affect the internal comparative validity of the classical and hybrid models but limits the extent to which the results represent a real-time forecasting scenario.

Future studies should therefore adopt a rolling-origin (walk-forward) or expanding-window validation scheme that respects chronological ordering. For example, a model could be trained on data from 2017–2018 and used to predict GCO outcomes for 2019, then retrained on 2017–2019 data to predict the year 2020, and so on. Averaging the year-ahead accuracy across these rolling windows would provide a more realistic assessment of how the model performs when applied to future, unseen periods. Such a temporally aligned validation framework will improve generalizability, avoid information leakage, and better reflect the decision-making environment of auditors assessing going-concern uncertainty.

## Supporting information

S1 Appendix Supplementary Tables. (DOCX)
