# Supplementary material for: Forecasting auditor’s going concern opinion using with hybrid robust machine learning model
Source: PLoS One. 2026 Mar 20;21(3):e0345071. doi: 10.1371/journal.pone.0345071 (PMC13004351; doi:10.1371/journal.pone.0345071)
Supplement: S1 Appendix — (DOCX) [file pone.0345071.s001.docx]

**Table A1.** Optimizing hyperparamteres of the machine learning models.

| **No** | **Feature selection** | **Machine learning models** | **Hyper Parameters** |
| --- | --- | --- | --- |
| **1** | **None** | **RF** | clf__n_estimators': 400, 'clf__min_samples_split': 3, 'clf__min_samples_leaf': 1, 'clf__max_features': 'sqrt', 'clf__max_depth': None |
|  |  | **XGB** | clf__subsample': 0.8, 'clf__reg_lambda': 1.7333333333333336, 'clf__reg_alpha': 0.8, 'clf__n_estimators': 1300, 'clf__min_child_weight': 2, 'clf__max_depth': 8, 'clf__learning_rate': 0.2355, 'clf__gamma': 0.5, 'clf__colsample_bytree': 0.5 |
|  |  | **KNN** | 'clf__weights': 'uniform', 'clf__p': 2, 'clf__n_neighbors': 9 |
|  |  | **GBM** | clf__subsample': 0.8, 'clf__n_estimators': 200, 'clf__max_depth': 4, 'clf__learning_rate': 0.1577 |
|  |  | **SVM** | 'clf__gamma': 'scale', 'clf__C': 7.8475 |
|  |  | **MLP** | 'clf__learning_rate_init': 0.01, 'clf__hidden_layer_sizes': (100, 50), 'clf__batch_size': 32, 'clf__alpha': 0.0129 |
| **2** | **GBM** | **RF** | 'clf__n_estimators': 200, 'clf__min_samples_split': 8, 'clf__min_samples_leaf': 7, 'clf__max_features': 'log2', 'clf__max_depth': 30 |
|  |  | **XGB** | clf__subsample': 0.5, 'clf__reg_lambda': 5.0, 'clf__reg_alpha': 0.0, 'clf__n_estimators': 900, 'clf__min_child_weight': 3, 'clf__max_depth': 3, 'clf__learning_rate': 0.074444, 'clf__gamma': 1.0, 'clf__colsample_bytree': 0.6 |
|  |  | **KNN** | 'clf__weights': 'uniform', 'clf__p': 1, 'clf__n_neighbors': 3 |
|  |  | **GBM** | clf__subsample': 0.9, 'clf__n_estimators': 400, 'clf__max_depth': 3, 'clf__learning_rate': 0.1155 |
|  |  | **SVM** | clf__gamma': 'scale', 'clf__C': 7.847 |
|  |  | **MLP** | clf__learning_rate_init': 0.000464, 'clf__hidden_layer_sizes': (200, 100), 'clf__batch_size': 32, 'clf__alpha': 0.004641 |
| **3** | **CART** | **RF** | RF ='clf__n_estimators': 400, 'clf__min_samples_split': 3, 'clf__min_samples_leaf': 1, 'clf__max_features': 'sqrt', 'clf__max_depth': None |
|  |  | **XGB** | GBM ='clf__subsample': 0.7, 'clf__n_estimators': 500, 'clf__max_depth': 4, 'clf__learning_rate': 0.1577777777777778 |
|  |  | **KNN** | XGB ='clf__subsample': 0.5, 'clf__reg_lambda': 5.0, 'clf__reg_alpha': 0.0, 'clf__n_estimators': 900, 'clf__min_child_weight': 3, 'clf__max_depth': 3, 'clf__learning_rate': 0.07444444444444444, 'clf__gamma': 1.0, 'clf__colsample_bytree': 0.6 |
|  |  | **GBM** | KNN ='clf__weights': 'distance', 'clf__p': 2, 'clf__n_neighbors': 3 |
|  |  | **SVM** | SVM ='clf__gamma': 0.03359818286283781, 'clf__C': 48.32930238571752 |
|  |  | **MLP** | MLP ='clf__learning_rate_init': 0.002154434690031882, 'clf__hidden_layer_sizes': (200, 100), 'clf__batch_size': 64, 'clf__alpha': 7.742636826811278e-05 |
| **4** | **Adaboost** | **RF** | RF ='clf__n_estimators': 900, 'clf__min_samples_split': 4, 'clf__min_samples_leaf': 1, 'clf__max_features': 'sqrt', 'clf__max_depth': 30 |
|  |  | **XGB** | GBM ='clf__subsample': 1.0, 'clf__n_estimators': 900, 'clf__max_depth': 2, 'clf__learning_rate': 0.07333333333333333 |
|  |  | **KNN** | XGB ='clf__subsample': 0.9, 'clf__reg_lambda': 4.455555555555556, 'clf__reg_alpha': 0.0, 'clf__n_estimators': 900, 'clf__min_child_weight': 2, 'clf__max_depth': 7, 'clf__learning_rate': 0.2677777777777778, 'clf__gamma': 2.5, 'clf__colsample_bytree': 0.9 |
|  |  | **GBM** | KNN ='clf__weights': 'distance', 'clf__p': 2, 'clf__n_neighbors': 5 |
|  |  | **SVM** | SVM ='clf__gamma': 0.08858667904100823, 'clf__C': 7.847599703514606 |
|  |  | **MLP** | MLP ='clf__learning_rate_init': 0.004641588833612777, 'clf__hidden_layer_sizes': (100, 50), 'clf__batch_size': 32, 'clf__alpha': 2.782559402207126e-05 |
| **5** | **RF** | **RF** | RF ='clf__n_estimators': 200, 'clf__min_samples_split': 10, 'clf__min_samples_leaf': 3, 'clf__max_features': 'log2', 'clf__max_depth': None |
|  |  | **XGB** | GBM ='clf__subsample': 0.9, 'clf__n_estimators': 700, 'clf__max_depth': 5, 'clf__learning_rate': 0.01 |
|  |  | **KNN** | XGB ='clf__subsample': 0.8, 'clf__reg_lambda': 0.1, 'clf__reg_alpha': 0.6000000000000001, 'clf__n_estimators': 900, 'clf__min_child_weight': 2, 'clf__max_depth': 7, 'clf__learning_rate': 0.1388888888888889, 'clf__gamma': 0.5, 'clf__colsample_bytree': 0.8 |
|  |  | **GBM** | KNN ='clf__weights': 'distance', 'clf__p': 2, 'clf__n_neighbors': 3 |
|  |  | **SVM** | SVM ='clf__gamma': 1.0, 'clf__C': 48.32930238571752 |
|  |  | **MLP** | MLP ='clf__learning_rate_init': 0.01, 'clf__hidden_layer_sizes': (200, 100), 'clf__batch_size': 32, 'clf__alpha': 0.0016681005372000592 |

**Table A2.** Detailed comparison of traditional and hybrid machine learning models in predicting Auditor opinion (%).

| No | Benchmark models | Type | Accuracy | F-1 Score | Precision | Recall | Base Model | Feature Selection |
| --- | --- | --- | --- | --- | --- | --- | --- | --- |
| 1 | Ada-RF | Hybrid | 0.940333 | 0.922201 | 0.940000 | 0.930440 | AdaBoost | RF |
| 2 | Ada-GBM | Hybrid | 0.940148 | 0.942201 | 0.919474 | 0.928839 | AdaBoost | GBM |
| 3 | None-XGB | Classic | 0.935708 | 0.948402 | 0.897895 | 0.921397 | None | XGB |
| 4 | CART-XGB | Hybrid | 0.923034 | 0.895974 | 0.929474 | 0.911905 | CART | XGB |
| 5 | Ada-XGB | Hybrid | 0.923127 | 0.921999 | 0.898947 | 0.909301 | AdaBoost | XGB |
| 6 | None-RF | Classic | 0.914524 | 0.886400 | 0.919474 | 0.901886 | None | RF |
| 7 | CART-RF | Hybrid | 0.906105 | 0.874532 | 0.910000 | 0.890944 | CART | RF |
| 8 | CART-GBM | Hybrid | 0.906105 | 0.889373 | 0.889474 | 0.888956 | CART | GBM |
| 9 | CART-MLP | Hybrid | 0.906105 | 0.900761 | 0.879474 | 0.887964 | CART | MLP |
| 10 | RF-RF | Hybrid | 0.901758 | 0.865714 | 0.909474 | 0.886829 | RF | RF |
| 11 | CART-KNN | Hybrid | 0.910176 | 0.922222 | 0.858947 | 0.886750 | CART | KNN |
| 12 | Ada-KNN | Hybrid | 0.905920 | 0.944444 | 0.828421 | 0.881903 | AdaBoost | KNN |
| 13 | RF-GBM | Hybrid | 0.897502 | 0.876036 | 0.890000 | 0.879713 | RF | GBM |
| 14 | GBM-RF | Hybrid | 0.893154 | 0.851190 | 0.910000 | 0.877964 | GBM | RF |
| 15 | None-SVM | Classic | 0.884459 | 0.834496 | 0.918947 | 0.872666 | None | SVM |
| 16 | GBM-XGB | Hybrid | 0.889177 | 0.866506 | 0.879474 | 0.871644 | GBM | XGB |
| 17 | CART-SVM | Hybrid | 0.888714 | 0.876625 | 0.858421 | 0.866607 | CART | SVM |
| 18 | None-GBM | Classic | 0.884459 | 0.864395 | 0.868947 | 0.865040 | None | GBM |
| 19 | RF-KNN | Hybrid | 0.884459 | 0.879564 | 0.848947 | 0.861620 | RF | KNN |
| 20 | Ada-SVM | Hybrid | 0.884459 | 0.924902 | 0.797895 | 0.854341 | AdaBoost | SVM |
| 21 | RF-MLP | Hybrid | 0.871600 | 0.827112 | 0.889474 | 0.854227 | RF | MLP |
| 22 | Ada-MLP | Hybrid | 0.876041 | 0.869986 | 0.838947 | 0.851528 | AdaBoost | MLP |
| 23 | GBM-KNN | Hybrid | 0.871693 | 0.857836 | 0.838421 | 0.847409 | GBM | KNN |
| 24 | GBM-GBM | Hybrid | 0.871970 | 0.859125 | 0.840000 | 0.846193 | GBM | GBM |
| 25 | GBM-SVM | Hybrid | 0.871785 | 0.872741 | 0.818421 | 0.843214 | GBM | SVM |
| 26 | None-MLP | Classic | 0.862442 | 0.846050 | 0.847368 | 0.843132 | None | MLP |
| 27 | RF-XGB | Hybrid | 0.867530 | 0.856358 | 0.828421 | 0.841361 | RF | XGB |
| 28 | GBM-MLP | Hybrid | 0.871878 | 0.886320 | 0.808947 | 0.840939 | GBM | MLP |
| 29 | RF-SVM | Hybrid | 0.863182 | 0.878006 | 0.787895 | 0.829453 | RF | SVM |
| 30 | None-KNN | Classic | 0.764492 | 0.754386 | 0.683333 | 0.702393 | None | KNN |
